# Successful bridge to heart transplantation in a pediatric patient with biventricular heart failure associated with Barth syndrome: a case report

**DOI:** 10.1007/s10047-026-01587-2

**Published:** 2026-09-08

**Authors:** Kazuto Arita, Takashi Kido, Masaki Taira, Takuji Watanabe, Jun Narita, Hidekazu Ishida, Ryo Ishii, Takayoshi Ueno, Shigeru Miyagawa

**Affiliations:** 1 Department of Cardiovascular Surgery, the University of Osaka Graduate School of Medicine, Osaka, Japan; 2 Department of Pediatrics, the University of Osaka Graduate School of Medicine, Osaka, Japan

**Keywords:** Barth syndrome, Heart transplantation, Biventricular assistance

## Abstract

Long waiting times for donor organs in pediatric patients have increased the need for mechanical circulatory support as a bridge to heart transplantation. We report the case of a one-year-old boy with Barth syndrome who presented with cardiogenic shock due to severe biventricular heart failure. Emergency biventricular assist device support using centrifugal pumps was initiated. Under severe pulmonary hypertension and pulmonary regurgitation, the right ventricular assist device outflow was temporarily directed to the left atrium to optimize left ventricular preload. As pulmonary hemodynamics improved, the right ventricular assist device outflow was redirected to the pulmonary artery, allowing evaluation of native pulmonary circulation and subsequent right ventricular assist device weaning. After recovery of end-organ function, the left ventricular assist device was converted to a Berlin Heart EXCOR 76 days after biventricular assist device implantation. The patient remained stable on Berlin Heart EXCOR support for 374 days and subsequently underwent successful heart transplantation. This case highlights the importance of early right ventricular assist device support and strategic management of right ventricular assist device outflow to optimize left ventricular assist device performance in pediatric patients with severe pulmonary hypertension. Such an approach may facilitate successful bridge-to-candidacy and eventual heart transplantation in high-risk pediatric patients.

## Introduction

The first pediatric heart transplantation in Japan was performed at the University of Osaka in 2011 [1]. By 2022, 68 patients aged < 18 years underwent heart transplantation in Japan. Although the overall survival rate after heart transplantation is 96.2% after 10 years in pediatric patients, the long waiting time of an average of 686 days for a donor organ represents a significant challenge, and the need for ventricular assist device (VAD) is increasing [2].

The Berlin Heart EXCOR (BHE) is the only durable VAD available for small children. In Japan, BHE was approved for clinical use in 2015 under the condition that it can only be used in patients who have completed heart transplant registration. Patients deemed suitable for heart transplantation are referred to certified centers where their candidacy is determined. The referred patients may have contraindications to transplant listing, including pulmonary hypertension, renal failure, and liver failure. Left ventricular assist device (LVAD) therapy is used to alleviate the comorbidities associated with heart failure and allows time for patients to improve their candidacy. This treatment strategy is called the bridge-to-candidacy (BTC) strategy.

Successful bridging treatment for heart transplant registration and subsequent BHE implantation is the primary challenge in managing pediatric end-stage heart failure. Here, we report our clinical experience with successful bridging treatment for BHE implantation and heart transplantation in a pediatric patient with Barth-associated dilated cardiomyopathy.

## Case report

A 1-year-old boy with severe heart failure due to Barth-associated dilated cardiomyopathy was transferred to our hospital. The patient was born at 40 weeks of gestation with a birth weight of 2608 g. The patient was admitted to a referral hospital at the age of 2 months because of poor weight gain and cardiac dysfunction. Oral medication was initiated, and the patient was discharged after a 2 month-hospital stay. Genetic testing revealed a nonsense mutation (c.153C > G) in the TAZ gene, suggesting that the patient may have Barth syndrome. The patient was readmitted to the referral hospital at the age of 1 year because of severe cardiac dysfunction with a brain natriuretic peptide (BNP) level of 2096 pg/mL. Despite the administration of inotropic agents, the patient’s clinical condition did not improve, and he was transferred to our hospital for heart transplant registration and BHE implantation.

**Fig. 1:**
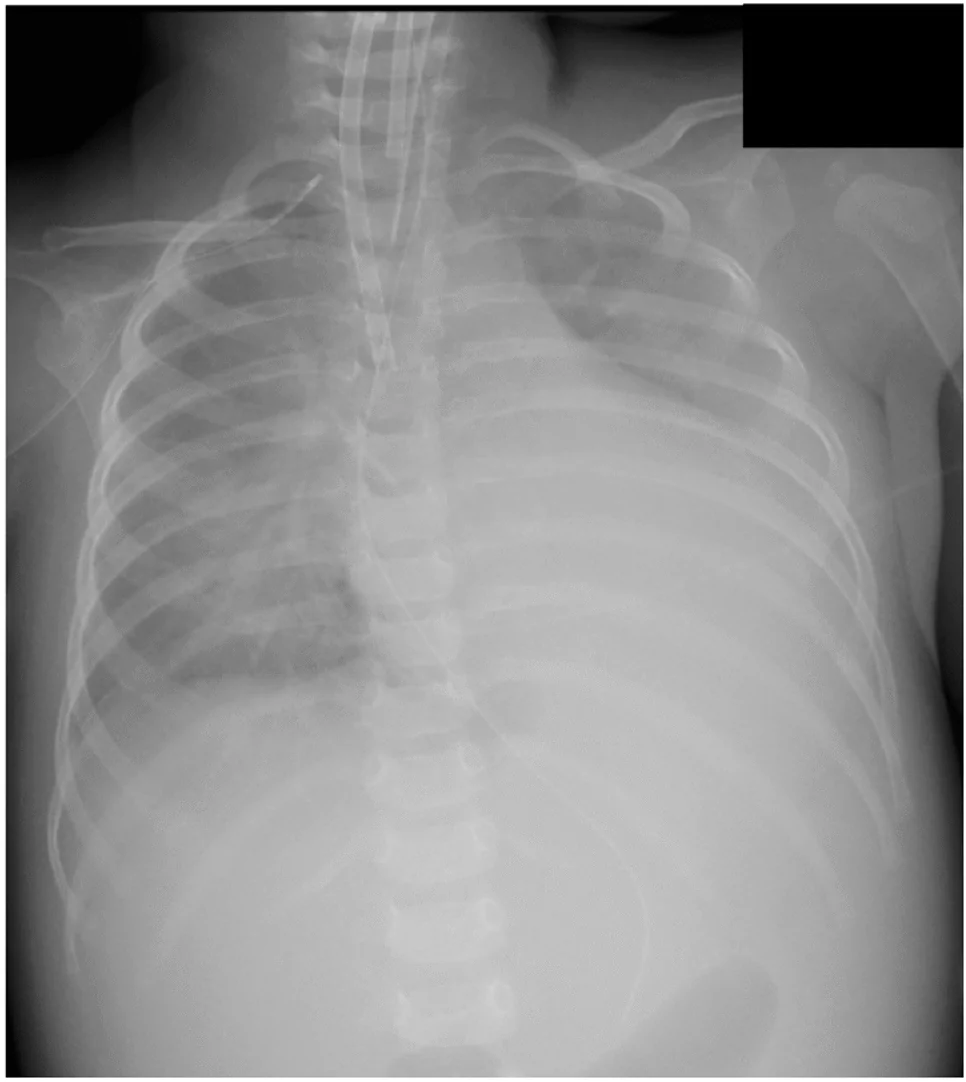
Chest X ray after ECMO establishment via neck vessels. ECMO, extracorporeal membrane oxygenation

On admission, the patient was 67.5 cm in height and weighed 6.1 kg. Echocardiography revealed severe cardiac dysfunction, with a left ventricular (LV) diastolic diameter of 38 mm (z-score: + 5.7), LV ejection fraction of 15%, and severe mitral regurgitation. The BNP level was 7667 pg/mL. After admission, the patient developed non-sustained ventricular tachycardia, and amiodarone therapy was initiated. Consequently, the patient’s blood pressure decreased, and emergent neck venoarterial extracorporeal membrane oxygenation was established. Subsequently, the patient developed acute pulmonary congestion, and emergency biventricular assist device (BiVAD) implantation was performed (Fig. 1). Centrifugal pumps were used for both ventricles, with an artificial lung in the LVAD circuit. For the LVAD cannula, we selected a 6-mm apex cannula (C18A-020) as the inflow cannula and a 6 mm arterial cannula (C19V-020) as the outflow cannula. Whereas, for the right VAD (RVAD), we inserted a 14-Fr inflow cannula (LARGE FLOW® VENOUS RETURN CANNULA) and an 8-Fr outflow cannula (Bio-Medicus® NextGen arterial cannula) into the inferior vena cava via the right atrium and distal pulmonary artery trunk, respectively, both with a purse-string suture. The RVAD functioned at a pumping flow rate of approximately 900 mL/min; however, the LVAD did not function efficiently, likely because of pulmonary hypertension and pulmonary regurgitation. The outflow cannula insertion site was replaced with the left atrium, and the LVAD flow rate was stabilized at approximately 600 mL/min.

The postoperative course was complicated by acute renal and liver failure, and continuous hemodiafiltration was initiated on postoperative day 7. Pulmonary congestion improved with biventricular support, and anti-pulmonary hypertension drugs were initiated. Cardiac catheterization was performed on postoperative day 5. During temporary interruption of RVAD flow, pulmonary artery pressure was 21/15 (mean 18) mmHg and pulmonary vascular resistance index was 3.5 WU m^2^, indicating acceptable pulmonary vascular resistance. Left atrial cannulation is associated with an increased risk of systemic thromboembolic events and does not allow removal of the artificial lung from the circuit. Therefore, the redirection of the RVAD outflow cannula from the left atrium to the pulmonary artery trunk, and removal of the artificial lung were performed on postoperative day 13. After conversion of the RVAD outflow cannula to the pulmonary artery, bedside echocardiography demonstrated improvement in right ventricular contraction, while pulmonary congestion progressed on chest X-ray. We considered that excessive pulmonary blood flow caused by RVAD support contributed to the pulmonary congestion. Therefore, RVAD flow was gradually reduced to provide low-flow support. During this period, LVAD flow remained stable at 900 mL/h and central venous pressure was maintained at approximately 12 mmHg, even when RVAD support was minimized to 400 mL/h or temporarily interrupted, suggesting adequate right ventricular function and pulmonary circulation to maintain LVAD preload. Considering the risk of circuit thrombosis associated with prolonged low-flow RVAD support and the stable hemodynamics without RVAD assistance, the RVAD was successfully removed on postoperative day 20.

**Fig. 2:**
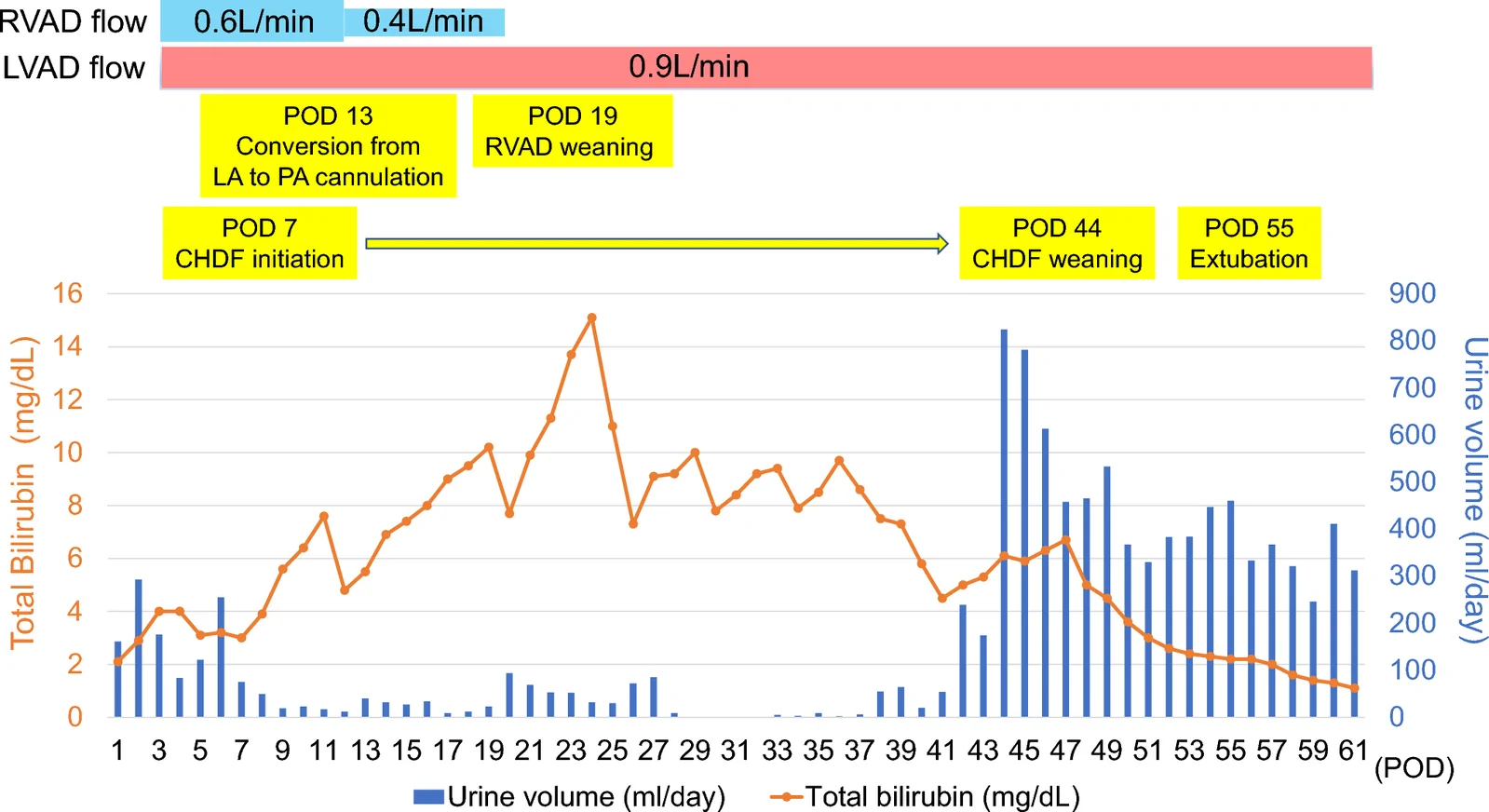
Treatment course in the Bridge-to-Candidacy strategy. CHDF, continuous hemodiafiltration; LA, left atrium; LVAD, left ventricular assist device; PA, pulmonary artery; POD, postoperative days; RVAD, right ventricular assist device

**Fig. 3:**
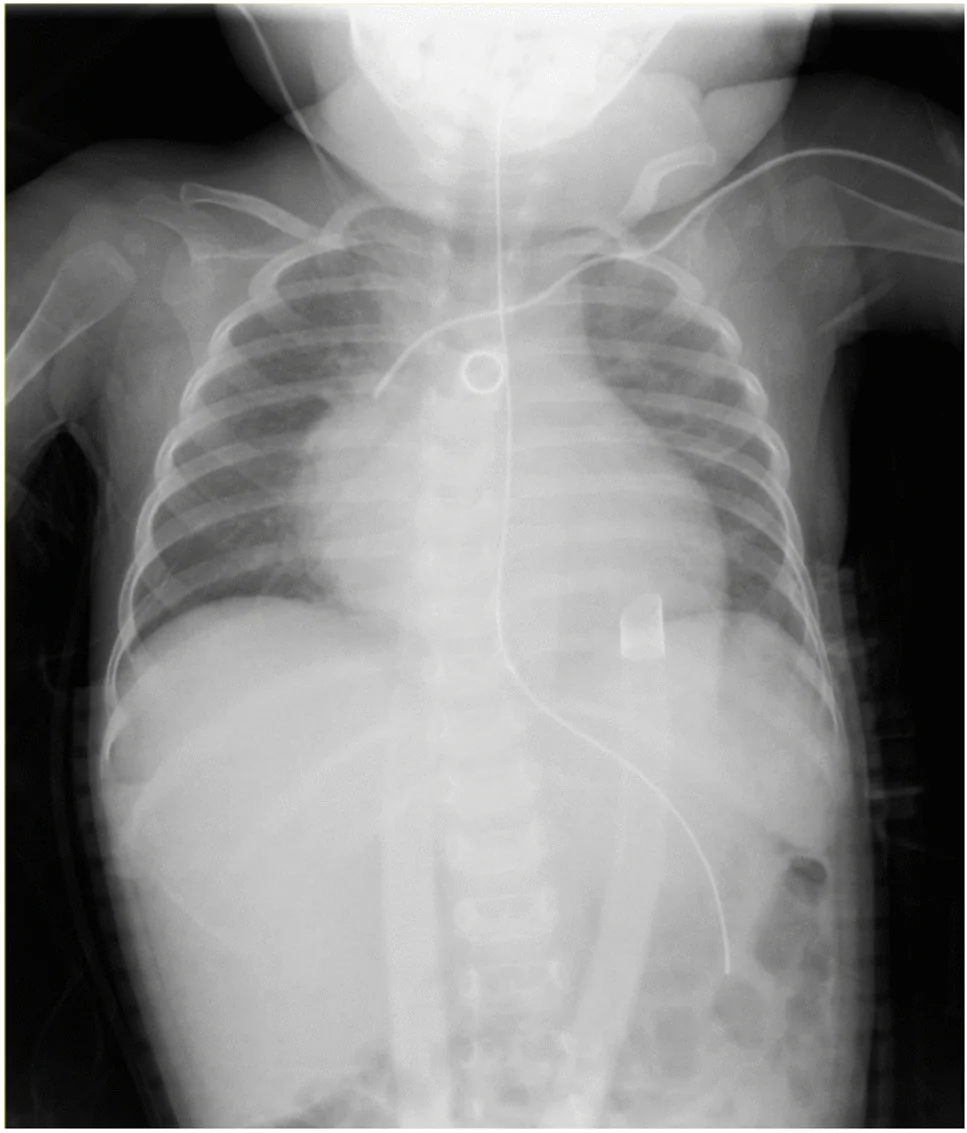
Chest X ray after Berlin Heart Excor implantation

Renal dysfunction and hyperbilirubinemia (maximum 15.1 mg/dL) persisted for more than a month; however, the patient was removed from continuous hemofiltration on postoperative day 44, and total bilirubin levels normalized on postoperative day 61. The patient was extubated on postoperative day 55. The treatment course for the BTC strategy is shown in Fig. 2. After obtaining in-house approval for heart transplant registration, the LVAD was converted to a BHE (pump size of 10 mL) 76 days after BiVAD implantation (Fig. 3). The patient remained stable during the waiting period and underwent successful heart transplantation after 374 days of BHE support.

## Discussion

### Bridge-to-candidacy strategy in pediatric patients with end-stage heart failure

Outcomes of BTC strategies have been widely investigated in adults. Yoshioka et al. reported their experience with LVAD implantation in 382 patients, including 45 with short-term mechanical circulatory support (MCS) as a BTC strategy for refractory cardiogenic shock [3]. Short-term MCS was used for a median of 14 days, improving end-organ function and hemodynamics and resulting in successful conversion to a durable VAD. Notably, 27% of BTC patients required RVAD support, which was identified as a significant factor associated with mortality. Shaw et al. analyzed the outcomes of durable LVAD as a BTC strategy using the UK national VAD database [4]. They reported that BTC is common in 51% of durable LVAD implants in the UK and is clinically effective, with survival and transplant incidence rates comparable to the bridge-to-transplant strategy.

In pediatric patients, BHE is the only durable VAD that can be used for small children. Since the use of BHE was first reported in 1991, outcomes after BHE implantation have been reported by several authors; however, there are limited data regarding BTC strategies in pediatric patients. A group in Berlin reported their 10-year-experience with 56 patients undergoing BHE implantation [5]. Ten of their cohorts were supported by ECMO or centrifugal VAD before BHE implantation, and the overall survival was similar to that of the bridge-to-transplant group. A multicenter US study demonstrated that low body weight, renal and hepatic dysfunction, and BiVAD support were associated factors with mortality, whereas ECMO before BHE implantation was not an associated factor [6]. This case report highlights the effectiveness of a BTC strategy with centrifugal VAD in a pediatric patient who presented with acute renal and liver failure secondary to dilated cardiomyopathy.

### Surgical considerations for heart transplantation in Barth syndrome

Barth syndrome is an X-linked recessive disorder characterized by dilated cardiomyopathy, muscular hypotonia, and cyclical neutropenia. The genetic basis of Barth syndrome involves mutations in the TAZ gene, which encodes a mitochondrial enzyme that remodels cardiolipin, a critical protein in normal mitochondrial structure and function. Because of the associated features of generalized myopathy and immunocompromise, patients with Barth syndrome are not considered suitable candidates for heart transplantation. Following the first successful heart transplantation reported by Adwani et al. in 1997, a group in London reported four Barth patients who had undergone successful heart transplantation [7, 8]. Based on these reports, heart transplantation has been recognized as an effective treatment for end-stage heart failure associated with Barth syndrome. Recently, Li et al. reported registry-based outcomes of heart transplantation in patients with Barth syndrome [9]. A total of 43 patients with Barth syndrome underwent heart transplantation. Among them, 29% were on VAD and 5% were on ECMO before heart transplantation. The outcomes after heart transplantation were similar in patients with and without Barth syndrome with regard to survival, infection, malignancy, and resolution of allograft vasculopathy. Although heart transplantation can effectively treat end-stage heart failure in patients with Barth syndrome, several systemic manifestations of the disease may persist after transplantation. Barth syndrome is characterized not only by cardiomyopathy but also by skeletal myopathy and mitochondrial metabolic abnormalities caused by defects in cardiolipin remodeling. Recent reports have suggested that heart transplantation may not fully normalize exercise tolerance, muscle mass, or substrate metabolism in these patients [10]. Therefore, even after successful heart transplantation, long-term multidisciplinary management and careful follow-up are required. In the present case, detailed evaluations of exercise capacity, skeletal muscle mass, and metabolic parameters were not systematically performed after transplantation, which represents a limitation of this report. Nevertheless, our case demonstrates that heart transplantation can be a life-saving treatment option for patients with Barth syndrome who develop end-stage heart failure, particularly when appropriate mechanical circulatory support strategies are applied before transplantation. Currently, the patient is 4 years old, two years after transplantation, and has achieved independent ambulation and verbal communication. Continued follow-up regarding neurodevelopmental progress and metabolic status remains ongoing.

### Strategic management of RVAD support

**Fig. 4:**
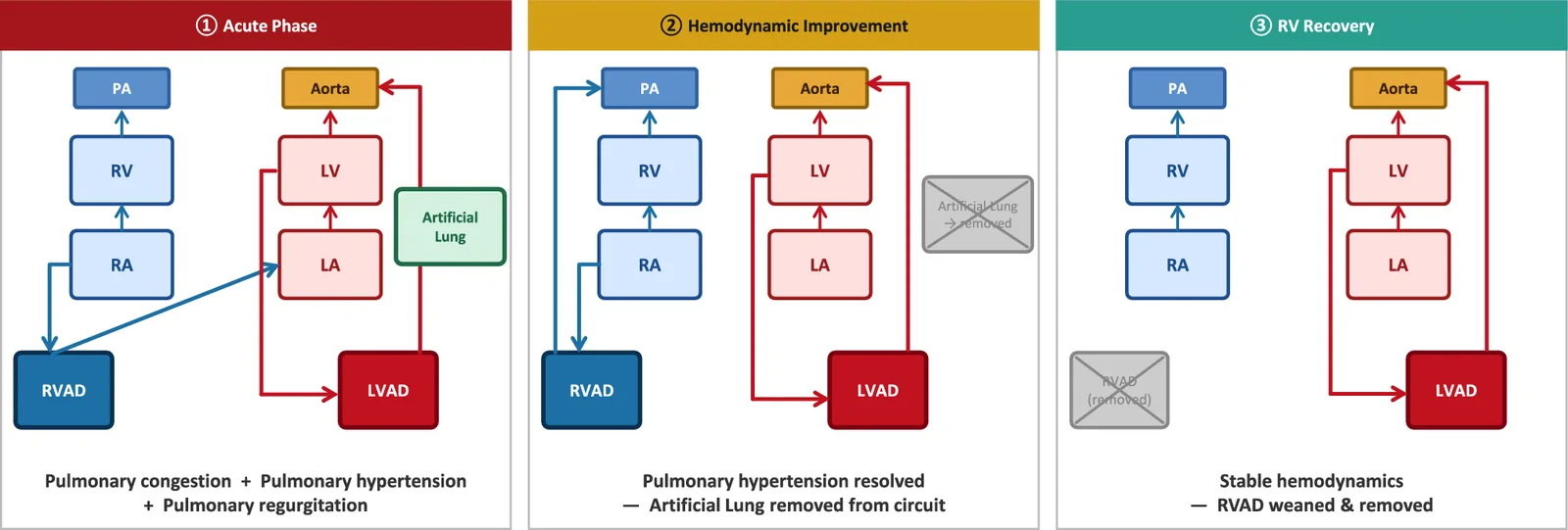
Strategy of RVAD Outflow Management. RVAD, right ventricular assist device

In patients with severe pulmonary hypertension and pulmonary regurgitation, conventional RVAD support directed to the pulmonary artery may lead to inefficient pulmonary circulation and inadequate LVAD preload. In our case, temporary diversion of the RVAD outflow to the left atrium allowed stabilization of LVAD flow during the acute phase (Fig. 4). As pulmonary congestion improved and pulmonary vascular resistance decreased, the RVAD outflow was redirected to the pulmonary artery to prevent systemic thromboembolic events and allow removal of the artificial lung from the circuit. This stepwise strategy enabled safe RVAD weaning while maintaining adequate systemic perfusion. This approach may be particularly useful in pediatric patients with biventricular failure complicated by pulmonary hypertension, where optimization of LVAD preload and careful evaluation of pulmonary circulation are critical for successful bridge-to-candidacy.

Detailed BTC strategies for patients with Barth syndrome have not yet been reported. Our patient presented with refractory cardiogenic shock and required centrifugal VAD as a BTC strategy for 78 days to improve pulmonary hypertension, renal failure, and liver failure. The patient underwent successful heart transplantation after 378 days of BHE support. The efficacy of BTC strategies in pediatric patients needs to be further elucidated to make appropriate decisions in the management of pediatric end-stage heart failure.
